# SOCIAL DETERMINANTS OF BRIDGE EMPLOYMENT: DO BIRTH COHORTS MATTER?

**DOI:** 10.1093/geroni/igad104.3593

**Published:** 2023-12-21

**Authors:** Gayoung Choi, Haena Lee

**Affiliations:** Sungkyunkwan University, Seoul, Republic of Korea; Sungkyunkwan University, Seoul, Republic of Korea

## Abstract

A growing number of retirees now engage in bridge employment, a term referring to their continued participation in the workforce post-retirement. The generation in South Korea born between 1945 and 1955, a period marked by leading industrialization and experiencing economic crises during their working age, exhibits unique socioeconomic characteristics distinct from later generations. Consequently, their retirement trajectories diverge from those of later cohorts, including the Baby Boomer generation (born between 1956 and 1965). In this paper, we investigate whether social, demographic, psychological, and health factors are associated with bridge employment and whether this association differs across birth cohorts. Data were drawn from seven waves of the Korean Longitudinal Study of Aging, conducted between 2008 and 2020. We employ an integrative framework that considers socioeconomic status, health status, and psychological factor s as the main variables to explain the odds of continuing to work after retirement. Retirement status includes three categories: employed, bridge, and total retirement. Results from a multinomial logit model reveal birth cohort differences in the relationship between social/psychological predictors and bridge employment. Additionally, higher educational attainment, a private pension, and poor mental health are associated with decisions regarding bridge employment for the industrialization generation, but not for Baby Boomers. Furthermore, females are more likely to participate in bridge employment than males for the industrialization generation, but not for Baby Boomers. Our findings contribute to the current literature on bridge employment by identifying the social determinants of bridge employment and highlighting the importance of cohort differences in bridge employment.

